# Incidence of Ectopic Pregnancies at a Tertiary Care Center of Tribal India: A Hospital-Based Cross-Sectional Study

**DOI:** 10.7759/cureus.92319

**Published:** 2025-09-14

**Authors:** Sona Singh, Vikas Gupta, Neha Jain, Shruti S Parihar, Ayushi Jaiswal, Kumari Snigda

**Affiliations:** 1 Obstetrics and Gynecology, Birsa Munda Government Medical College, Shahdol, IND; 2 Community Medicine, Government Medical College, Seoni, IND; 3 Obstetrics and Gynecology, Government Medical College, Satna, IND

**Keywords:** ectopic pregnancy, maternal morbidity, ruptured ectopic, tertiary care, tribal population

## Abstract

Background and aim

Ectopic pregnancy remains a significant cause of maternal morbidity and mortality, particularly in low-resource settings where delayed diagnosis and limited healthcare access exacerbate adverse outcomes. The burden is even more pronounced among tribal populations due to systemic healthcare barriers. This study aimed to evaluate the clinical profile, risk factors, treatment modalities, and maternal outcomes of ectopic pregnancies managed at a tertiary care hospital catering to a predominantly tribal population in central India.

Methods

A hospital-based cross-sectional study was conducted at Birsa Munda Government Medical College (BMGMC), Shahdol, a tertiary healthcare facility in Madhya Pradesh, India, from February 2024 to January 2025. Pregnant women diagnosed with ectopic pregnancy based on clinical symptoms, transvaginal ultrasonography (TVS), and serum beta-human chorionic gonadotropin (β-hCG) assays were included. Comprehensive data on demographics, obstetric history, clinical presentation, laboratory findings, treatment modalities, and surgical outcomes were collected. Statistical analysis was performed using SPSS version 20.0 (Armonk, NY: IBM Corp.), with a p<0.05 considered statistically significant.

Results

A total of 92 ectopic pregnancy cases were analyzed. Abdominal pain (97.8%) and amenorrhea (95.7%) were the most common symptoms, with 66.4% presenting after 24 hours. The vast majority of cases were tubal (97.8%), with the ampullary segment being the most commonly affected site (71.7%). Ruptured ectopic pregnancy occurred in 84.8% of cases. Delayed presentation (OR: 9.82, 95% CI: 2.32-41.54; p=0.002), hemoglobin <10 g/dL (OR: 10.54, 95% CI: 2.94-37.82; p<0.001), and β-hCG >5000 mIU/mL (OR: 10.01, 95% CI: 2.06-48.69; p=0.004) were significantly associated with rupture. All cases required surgical intervention, with unilateral salpingectomy being the most common procedure (43.5%). The mean hospital stay was 4.8±2.1 days, and 28.3% required ICU care. No maternal mortality was reported.

Conclusion

This study underscores the high burden of ruptured ectopic pregnancies in tribal regions, driven by delays in presentation and limited healthcare access. Early diagnosis, improved primary care referral systems, and targeted community awareness programs are urgently needed to reduce preventable maternal morbidity in underserved populations.

## Introduction

Ectopic pregnancy (EP) is defined as the implantation of a fertilized ovum outside the endometrial cavity of the uterus. The fallopian tube is the most frequent site, accounting for approximately 95-98% of cases, particularly the ampullary region. Less common sites include the ovary (1-3%), cervix (<1%), and the abdominal cavity (<1%) [1]. EP constitutes a significant obstetric emergency and remains a major contributor to maternal morbidity and mortality globally, particularly in low-resource settings where access to timely diagnostic and surgical care is limited.

Globally, EP occurs in about 1-2% of all pregnancies, but it is responsible for a disproportionately high percentage of pregnancy-related deaths, estimated at 6-9%, primarily due to delayed diagnosis and inadequate access to emergency care [2]. In India, the incidence of EP ranges from 1% to 2.5%, with considerable regional variability influenced by disparities in healthcare infrastructure, socioeconomic status, and reproductive health literacy [3]. Among these, tribal communities represent a particularly vulnerable group, facing heightened risks due to late presentation, limited diagnostic availability, sociocultural barriers, and reliance on traditional healers [4].

Multiple risk factors have been implicated in the pathogenesis of EP, most notably pelvic inflammatory disease (PID), prior tubal surgery, previous ectopic pregnancy, intrauterine contraceptive device (IUCD) usage, infertility treatments, and structural abnormalities of the fallopian tubes [5]. Indian studies have reported a 30-40% association between EP and previous PID, highlighting the role of untreated or recurrent reproductive tract infections in tubal damage [6]. This is especially relevant in underserved populations, where barriers to timely STI diagnosis and treatment are prevalent.

A particularly alarming trend is the delay in diagnosis and care among tribal and rural populations. Rupture rates among tribal women with EP are reportedly as high as 60-70%, in stark contrast to 30-40% in urban settings, primarily due to late presentation and inadequate antenatal care [7]. Complications such as hemorrhagic shock, hemodynamic instability, and the need for emergency surgical interventions, including salpingectomy, are frequently observed. A recent Indian report noted that over four-fifths of women with EP presented in a critical condition, half required salpingectomy, and nearly one-fifth were hemodynamically unstable upon admission [8]. Despite technological advancements, including widespread use of transvaginal ultrasonography and serum beta-human chorionic gonadotropin (β-hCG) testing, the benefits of early detection are not uniformly realized in rural and tribal regions due to logistical and systemic barriers [9].

Moreover, the scarcity of trained personnel, poor antenatal care utilization, and continued preference for traditional medicine over institutional care further exacerbate delays in diagnosis and management. These factors collectively underscore the urgent need for population-specific data to inform targeted interventions in high-risk communities [10,11].

Given these challenges, this hospital-based cross-sectional study was conducted at a tertiary care center in central India serving a predominantly tribal population. The aim of the study was to evaluate the incidence, clinical presentation, risk factors, and outcomes associated with ectopic pregnancies in this demographic. Insights from this study are expected to inform early detection strategies, guide community-level interventions, and ultimately reduce maternal morbidity and mortality related to ectopic pregnancy in underserved regions.

## Materials and methods

Study design and setting

This cross-sectional observational study was conducted over a one-year period, from February 2024 to January 2025, in the Department of Obstetrics and Gynecology at Bundelkhand Medical Government Medical College (BMGMC), Shahdol, Madhya Pradesh, India. BMGMC serves as a tertiary care and referral center for a predominantly tribal and rural population across central India, where healthcare access remains limited. During the study period, approximately 2550 deliveries and 3100 obstetric admissions were recorded, providing critical context to the burden and incidence of ectopic pregnancy within the institution’s catchment area. The primary aim of the study was to evaluate the clinical profile, risk factors, diagnostic modalities, management strategies, and immediate maternal outcomes associated with ectopic pregnancies.

Study population

All pregnant women diagnosed with ectopic pregnancy during the study period were included in the study. The diagnosis of ectopic pregnancy was confirmed through a standardized protocol that integrated clinical evaluation, transvaginal ultrasonography (TVS), and measurement of serum β-human chorionic gonadotropin (β-hCG) levels. Patients were excluded if their clinical records were incomplete, if they had undergone surgical intervention at another institution prior to presentation, or if they declined to participate after counseling and consent. In cases with equivocal findings, repeat β-hCG testing and serial TVS imaging were performed to ensure diagnostic accuracy and minimize misclassification.

Diagnostic criteria

All patients underwent a structured diagnostic approach. Clinical evaluation focused on symptoms such as abdominal pain, vaginal bleeding, and signs of hemodynamic instability. TVS was utilized to identify the presence of an adnexal mass, an extrauterine gestational sac, or free pelvic fluid. Serum β-hCG levels were interpreted in conjunction with sonographic findings. A β-hCG discriminatory threshold of 1500-2000 mIU/mL was applied, whereby the absence of an intrauterine gestational sac above this range raised a high suspicion for ectopic pregnancy. In addition, a plateau or suboptimal rise in β-hCG levels over a 48-hour period was considered suggestive of a non-viable pregnancy, including ectopic gestation.

Sample size and sampling technique

The sample size was estimated using a standard prevalence-based formula: n=Z^2^×P(1−P)/d^2^, where Z represents the standard normal variate (1.96 for 95% confidence), P represents the expected prevalence (2% based on national data), and d represents the absolute precision (2%). Based on this calculation, a minimum sample size of 76 was obtained [2]. Accounting for a 10% margin to compensate for exclusions or incomplete data, the final target sample size was adjusted to 82 cases. Ultimately, 92 patients fulfilling the inclusion criteria were enrolled during the study period. A universal sampling technique was employed, in which all eligible and consenting cases of ectopic pregnancy were consecutively included.

Data collection

Data were collected prospectively using a structured questionnaire. Information on demographic variables such as age, socioeconomic status (classified according to the modified BG Prasad scale), and educational attainment was recorded. Detailed obstetric and gynecological history was obtained, including gravidity, parity, history of previous ectopic pregnancy, prior cesarean section, pelvic inflammatory disease, tubal surgeries (such as sterilization procedures), and use of assisted reproductive technologies or infertility treatments. Clinical presentations were systematically documented, encompassing abdominal pain, vaginal bleeding, amenorrhea, dizziness, and episodes of syncope. General physical and systemic examinations were conducted, with a focus on assessing hemodynamic stability, abdominal tenderness, adnexal masses, and peritoneal signs.

Diagnostic findings included transvaginal sonographic features of the ectopic pregnancy, serum β-hCG levels, and laboratory investigations such as hemoglobin concentration, with anemia categorized according to the World Health Organization criteria into mild, moderate, or severe. Patients were stratified based on hemodynamic status at presentation to inform management decisions.

Management and outcome assessment

**Figure 1:**
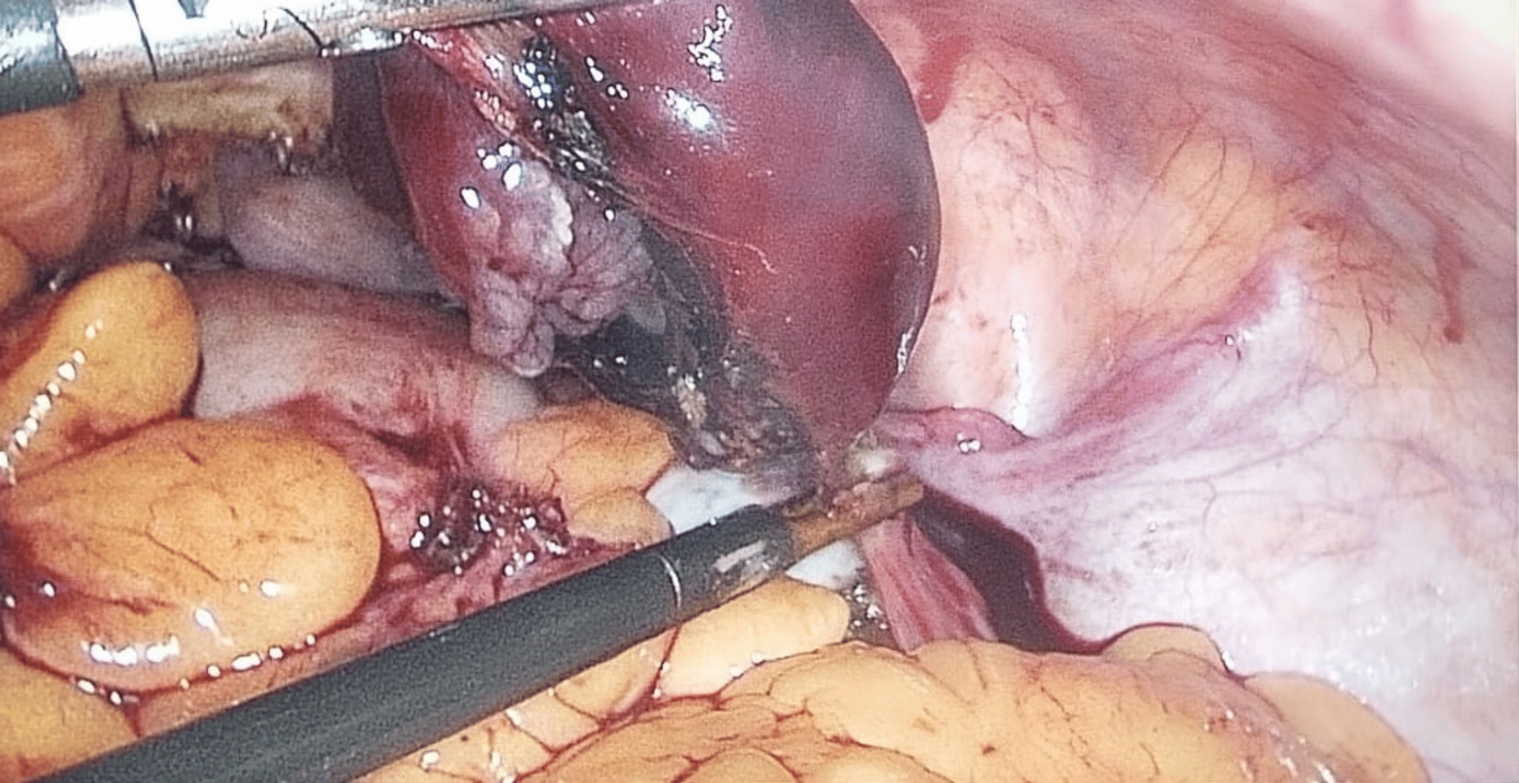
Laparoscopic view of ruptured tubal ectopic pregnancy - left ampullary region.

**Figure 2:**
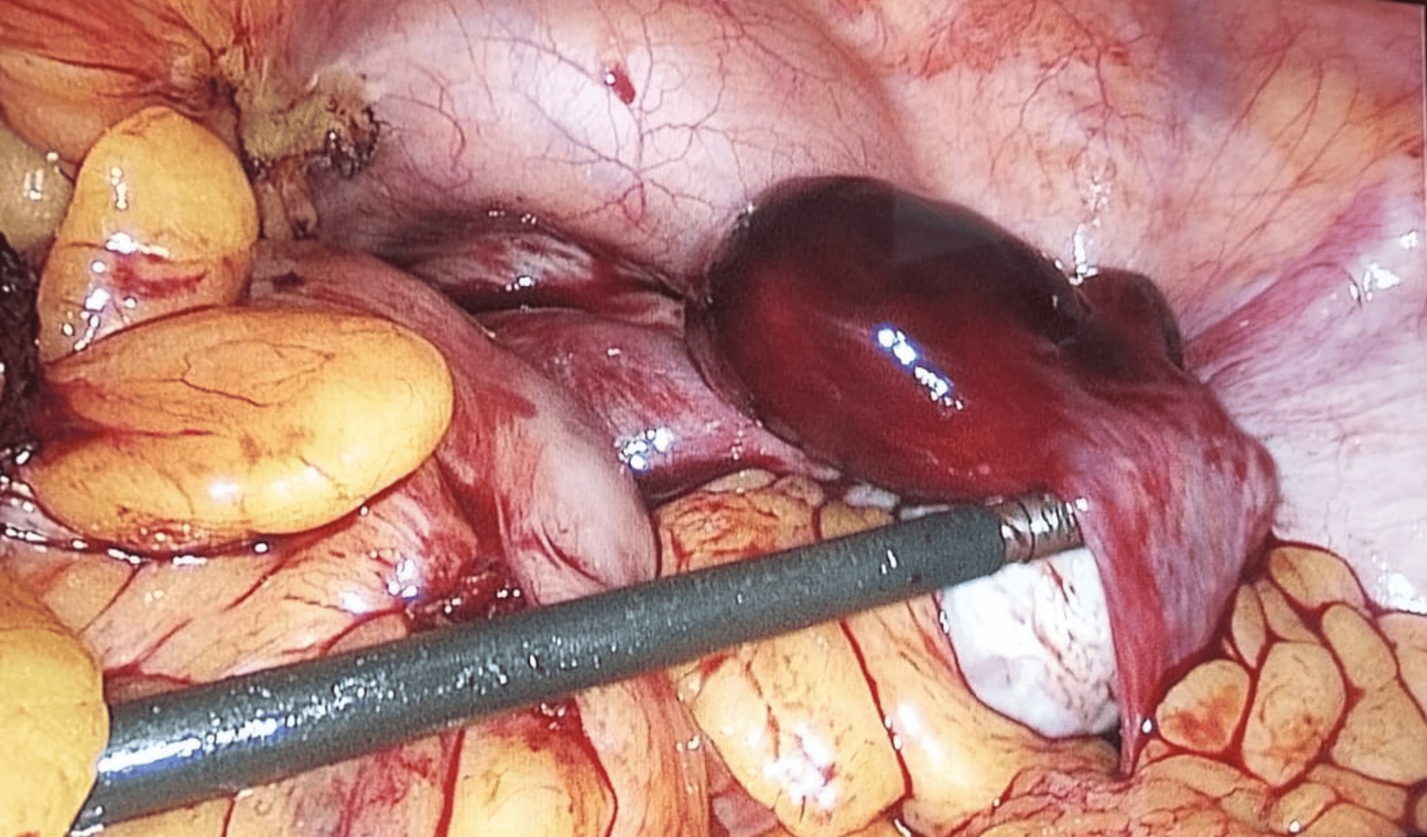
Laparoscopic view of ruptured tubal ectopic pregnancy - right ampullary region.

All cases were managed surgically, with the choice of procedure guided by the patient’s clinical status and intraoperative findings. Surgical interventions included unilateral or bilateral salpingectomy, salpingo-oophorectomy, or, where appropriate, conservative tubal surgeries (Figures 1, 2). The volume of hemoperitoneum encountered intraoperatively, the presence or absence of tubal rupture, the condition of the contralateral fallopian tube, and the need for intraoperative or postoperative blood transfusions were meticulously recorded. Immediate postoperative outcomes were assessed during the hospital stay, including the duration of hospitalization, occurrence of surgical site infections, wound dehiscence, admission to intensive care units, requirement for re-exploration, and maternal mortality.

Statistical analysis

All data were entered into Microsoft Excel and analyzed using IBM SPSS Statistics version 20.0 (Armonk, NY: IBM Corp.) for Windows. Categorical variables were presented as frequencies and percentages, whereas continuous variables were expressed as means with standard deviations. Differences between ruptured and unruptured ectopic pregnancies were analyzed using the chi-square test or Fisher’s exact test, depending on the distribution of data. Logistic regression analysis was performed to identify independent predictors of tubal rupture. Variables entered into the multivariable model included socioeconomic status, education level, rural residence, history of previous ectopic pregnancy, history of pelvic inflammatory disease, prior tubal surgeries, delayed presentation beyond 24 hours, presence of anemia, and initial β-hCG levels. A p-value less than 0.05 was considered statistically significant.

Ethical considerations

The study was conducted in accordance with ethical standards and was approved by the Institutional Ethics and Review Committee (IEC) of BMGMC Shahdol (IEC no.: BMGMC/IERC/2023/48, dated 05/01/2024). Written informed consent was obtained from all participants prior to enrollment. Patient confidentiality and privacy were strictly maintained through anonymization of data and secure storage protocols. The study adhered to the principles of the Declaration of Helsinki (2013 revision), ensuring that participation did not influence the standard of clinical care provided to patients.

## Results

During the one-year study period, a total of 2,543 deliveries were conducted at the tertiary care center, and 92 cases of ectopic pregnancy were diagnosed, yielding a hospital-based incidence rate of 3.62%. The mean age of the study participants was 27.9±5.8 years. A majority of the women (63.0%) were within the 20-30 years age group, 10.9% were younger than 20 years, and 26.1% were older than 30 years. Regarding marital status, 96.7% of the participants were married, while a minority were unmarried (2.2%) or separated/divorced (1.1%). Socioeconomic classification was performed using the Modified BG Prasad Socioeconomic Classification (2024 update). Based on this scale, a significant proportion of participants (71.7%) belonged to the lower socioeconomic class, 23.9% were from the middle class, and 4.4% were from the upper class. In terms of educational attainment, 32.6% of women had no formal education, 30.4% had completed primary education, 26.1% had attained secondary education, and only 10.9% had received education beyond the secondary level. Regarding current occupational status, 70.6% of the women were categorized as homemakers - defined as individuals actively engaged in domestic responsibilities without seeking paid employment outside the home - while 19.6% were classified as unemployed, referring to those neither engaged in domestic duties nor any form of paid work. Only 9.8% of the participants were employed outside the home in various occupations. The study population was predominantly rural, with 87.0% of the participants residing in rural areas and only 13.0% residing in urban locations (Table 1).

**Table 1:** Sociodemographic characteristics of ectopic pregnancy cases (n=92).

Variable	Frequency (%)
Age (years) (mean±SD=27.9±5.8)	
<20	10 (10.9)
20-30	58 (63.0)
>30	24 (26.1)
Marital status	
Married	89 (96.7)
Unmarried	2 (2.2)
Separated/divorced	1 (1.1)
Socioeconomic status (modified BG Prasad scale)	
Lower/lower middle class (per capita income)	66 (71.7)
Middle class (per capita income Rs. 2739-4564)	22 (23.9)
Upper/upper middle class (per capita income >Rs. 4564)	4 (4.4)
Educational level	
No formal education	30 (32.6)
Primary education	28 (30.4)
Secondary education	24 (26.1)
Higher education	10 (10.9)
Employment status	
Employed	9 (9.8)
Unemployed	18 (19.6)
Homemaker	65 (70.6)
Residence	
Rural	80 (87.0)
Urban	12 (13.0)

Of the 92 participants, the mean gravidity was 2.9±1.6, with 72.8% being multigravida and the remainder primigravida. The mean parity was 1.5±1.2; 23.9% were nulliparous, while 39.1% had experienced one prior delivery. Regular menstrual cycles were reported by 76.1% of participants, whereas 23.9% had irregular cycles. A prior history of miscarriage was present in 19.6% of the women, and 5.4% reported a previous ectopic pregnancy. A diagnosis of pelvic inflammatory disease (PID) was documented in 8.6% of participants. Regarding surgical history, 21.7% of women had undergone prior tubal surgery. Among them, 15.2% had a history of laparoscopic tubal ligation (tubal sterilization) conducted before the current ectopic pregnancy event. A history of previous lower-segment cesarean section (LSCS) was found in 10.9% of cases, and 5.4% reported undergoing infertility treatments (Table 2).

**Table 2:** Obstetric and gynecological history of ectopic pregnancy cases (n=92). LSCS: lower-segment cesarean section

Variable	Frequency (%)
Gravidity (mean±SD=2.9±1.6)	
Primigravida	25 (27.2)
Multigravida	67 (72.8)
Parity (mean±SD=1.5±1.2)	
Nulliparous	22 (23.9)
Para 1	36 (39.1)
Para 2	20 (21.7)
≥3	14 (15.3)
Menstrual cycle	
Regular	70 (76.1)
Irregular	22 (23.9)
Number of miscarriages	0.6±0.9
History of previous miscarriage	
Yes	18 (19.6)
No	74 (80.4)
History of previous ectopic pregnancy	
Yes	5 (5.4)
No	87 (94.6)
History of pelvic inflammatory disease (PID)	8 (8.6)
History of tubal surgery	
No	72 (78.3)
Laparoscopic tubal ligation (TT)	14 (15.2)
Abdominal tubal ligation (TT)	6 (6.5)
History of previous LSCS	10 (10.9)
History of infertility treatment	
Yes	5 (5.4)
No	87 (94.6)

Among the 92 patients diagnosed with ectopic pregnancy, the most common presenting symptoms were abdominal pain, reported in 97.8% of cases, and amenorrhea, observed in 95.7%. Vaginal bleeding was present in 47.8% of patients, while dizziness or syncope was reported by 19.6%, and shoulder tip pain by 9.8%. Clinical presentation in shock was noted in 13.0% of cases. The mean duration of symptoms before seeking care was 6.2±3.4 days, with delayed presentation (>24 hours after symptom onset) observed in 66.4% of patients. The mean hemoglobin level at presentation was 9.8±2.1 g/dL, with 77.2% of women presenting with moderate or severe anemia as per WHO classification. Serum β-hCG levels were elevated in the majority, with a detailed distribution as follows: 19.6% had β-hCG levels <1000 mIU/mL, 23.9% had levels between 1000 mIU/mL and 5000 mIU/mL, and 56.5% had levels >5000 mIU/mL. Transvaginal sonography (TVS) findings showed that 63.0% of patients had an adnexal mass without a visible gestational sac, while 45.7% demonstrated a definitive extrauterine gestational sac, and 41.3% had sonographic evidence of hemoperitoneum. Laterality assessment revealed that 45.7% of ectopic pregnancies involved the right fallopian tube, while 54.3% involved the left tube. Regarding the type of ectopic pregnancy, 97.8% were tubal pregnancies, with ampullary location being the most common (71.7%), followed by isthmic and fimbrial locations. Intraoperative findings revealed that 84.8% of cases were ruptured ectopic pregnancies at the time of surgical exploration (Table 3).

**Table 3:** Clinical presentation, and diagnostic parameters of ectopic pregnancy cases (n=92). β-hCG: beta-human chorionic gonadotropin

Variables	Frequency (%)
Clinical feature	
Abdominal pain	90 (97.8)
Vaginal bleeding	44 (47.8)
Amenorrhea (missed period)	88 (95.7)
Dizziness/syncope	18 (19.6)
Shoulder tip pain	9 (9.8)
Shock	12 (13.0)
Duration of symptoms (in days) (mean±SD=6.2±3.4)	
Presentation	
Early	31 (33.6)
Delayed	61 (66.4)
Diagnostic parameter	
Hemoglobin (g/dL) (mean±SD=9.8±2.1)	
Anemia	
Mild (hemoglobin=10-11 g/dL)	21 (22.8)
Moderate/severe (hemoglobin <10 g/dL)	71 (77.2)
Positive urine pregnancy test	92 (100.0)
Serum β-hCG level (mIU/mL) (mean±SD=4580.9±3212.8)	
Serum β-hCG level (mIU/mL)	
≤5000 mIU/mL	40 (43.5)
>5000 mIU/mL	52 (56.5)
Transvaginal ultrasound (TVS) findings	92 (100.0)
Adnexal mass without gestational sac	58 (63.0)
Extrauterine gestational sac	42 (45.7)
Hemoperitoneum	38 (41.3)
Free fluid in pouch of Douglas	60 (65.2)
Fetal cardiac activity present	12 (13.0)
Complex mass with echogenicity	34 (37.0)
Side of ectopic pregnancy	
Right	40 (45.7)
Left	52 (54.3)
Location of ectopic pregnancy	
Tubal	90 (97.8)
Ampullary	66 (71.7)
Isthmus	20 (21.8)
Cornua	4 (4.3)
Ovarian	2 (2.2)
Status of ectopic pregnancy (intraoperative)	
Ruptured	78 (84.8)
Not ruptured	14 (15.2)

Surgical management was performed in all cases, with unilateral salpingectomy being the most common procedure (43.5%), followed by unilateral salpingectomy with opposite-side tubectomy (32.6%) and bilateral salpingectomy (13.0%). Hemoperitoneum greater than 200 mL was observed in 30.4% of patients, which may have contributed to the need for more intensive surgical intervention. ICU admission was required for 28.3% of patients. Blood transfusion was necessary for 69.6% of patients due to severe anemia (Hb <7 g/dL). Postoperative infections and wound dehiscence occurred in 2.2% and 1.1% of cases, respectively. Re-exploration surgery was required in 3.3% of patients. The mean hospital stay was 4.8±2.1 days, with a mean time to mobilization of 16.4±6.3 hours. No mortality was reported (Table 4).

**Table 4:** Treatment modalities and clinical outcomes in ectopic pregnancy cases (n=92). *Others include B/L salpingectomy + U/L oophorectomy (n=3), U/L salpingectomy + opposite-side tubectomy + U/L oophorectomy (n=2), U/L salpingo-oophorectomy (n=2), exploratory laparotomy followed by hemoperitoneum removal for tubal abortion (n=2), and U/L salpingo-oophorectomy + U/L ovarian cystectomy (n=1). B/L: bilateral; U/L: unilateral; Hb: hemoglobin; ICU: intensive care unit

Treatment modality and outcome	Frequency (%)
Surgical management	
Unilateral salpingectomy + opposite-side tubectomy	30 (32.6)
Unilateral salpingectomy	40 (43.5)
Bilateral salpingectomy	12 (13.0)
Others*	10 (10.9)
Outcome parameters	
Hemoperitoneum >200 mL	28 (30.4)
ICU admission	26 (28.3)
Postoperative infections	2 (2.2)
Blood transfusion (Hb <7 g/dL)	64 (69.6)
Wound dehiscence	1 (1.1)
Deep vein thrombosis	0 (0.0)
Need for re-exploration surgery	3 (3.3)
Duration of hospital stay (days) (mean±SD=4.8±2.1)	
Time to mobilization (hours) (mean±SD=16.4±6.3)	
Mortality	0 (0.0)

Among the 92 cases, 78 (84.8%) had ruptured ectopic pregnancies. A lower socioeconomic status was significantly associated with rupture (AOR: 4.29, 95% CI: 1.10-16.75, p=0.035), as was delayed presentation greater than 24 hours (AOR: 10.63, 95% CI: 2.69-42.02, p=0.002). Hemoglobin levels below 10 g/dL (AOR: 9.90, 95% CI: 2.82-34.71, p<0.001) and serum β-hCG levels greater than 5000 mIU/mL (AOR: 10.71, 95% CI: 2.24-51.34, p=0.004) were also strongly associated with rupture. However, factors such as previous ectopic pregnancy, a history of pelvic inflammatory disease (PID), and tubal surgery were not statistically significant (Table 5).

**Table 5:** Predictors of ruptured versus unruptured ectopic pregnancy cases (n=92). *Statistically significant values. PID: pelvic inflammatory disease; β-hCG: beta-human chorionic gonadotropin; OR: odds ratio

Predictor variable	Ruptured (n=78)	Unruptured (n=14)	Adjusted OR (95% CI)	p-Value
Lower socioeconomic status	60 (76.9%)	6 (42.9%)	4.29 (1.10-16.75)	0.035*
No formal education	28 (35.9%)	2 (14.3%)	3.39 (0.66-17.45)	0.143
Rural residence	70 (89.7%)	10 (71.4%)	3.69 (0.72-18.98)	0.114
History of PID	7 (9.0%)	1 (7.1%)	1.28 (0.15-11.31)	0.813
Previous ectopic pregnancy	4 (5.1%)	1 (7.1%)	0.70 (0.07-6.80)	0.729
History of tubal surgery	18 (23.1%)	2 (14.3%)	1.80 (0.35-9.24)	0.478
Delayed presentation	58 (74.4%)	3 (21.4%)	10.63 (2.69-42.02)	0.002*
Hemoglobin <10 g/dL	66 (84.6%)	5 (35.7%)	9.90 (2.82-34.71)	<0.001*
Serum β-hCG >5000 mIU/mL	50 (64.1%)	2 (14.3%)	10.71 (2.24-51.34)	0.004*

## Discussion

This study provides a comprehensive analysis of ectopic pregnancies (EP) in a tertiary care center catering to a predominantly tribal population in India, emphasizing the high burden of ruptured ectopic pregnancies and associated risk factors. Our findings highlight critical gaps in early diagnosis and timely intervention, with significant implications for maternal morbidity.

During the study period, a total of 2543 deliveries were conducted at the tertiary care center, out of which 92 cases were diagnosed as ectopic pregnancies. This corresponds to an incidence rate of 3.62%, which is notably higher than the national average reported in previous Indian studies [12-17].

A notably high proportion (84.8%) of ectopic pregnancies in our study presented as ruptured, which is considerably higher than rates reported in studies by Ahirwar et al. and Gothwal and Pathak, from tertiary care centers in India, where rupture rates range between 50% and 65% [12,13]. This discrepancy likely stems from delayed healthcare access, limited awareness of early symptoms, and suboptimal antenatal care in rural and tribal populations. In the present study, patients had to travel an average distance of approximately 60 kilometers to reach the tertiary care center; however, due to hilly terrain, poor road conditions, and extremely limited transportation facilities, with bus services operating only once every 12 hours for many destinations, the journey often took 3 to 4 hours. These logistical barriers significantly contributed to delays in diagnosis and treatment, thereby influencing the higher rates of complications observed. Delayed presentation beyond 24 hours was identified as a major risk factor for rupture (OR: 9.82, 95% CI: 2.32-41.54, p=0.002), underscoring the logistical challenges in timely medical access. Similar trends have been reported in other Indian studies by Andola et al. and Verma et al., where systemic delays, including lack of transportation and financial constraints, contribute to adverse outcomes [14,15].

The clinical presentation of EP in our study was consistent with classical symptoms reported in the literature, with abdominal pain (97.8%), amenorrhea (95.7%), and vaginal bleeding (47.8%) being the most common complaints. These findings align with studies by Soren et al. and Barik et al., which emphasized that while the classic triad is a hallmark of EP, atypical presentations such as dizziness (19.6%) and shoulder tip pain (9.8%) should not be overlooked [16,17]. Notably, the presence of hemodynamic instability was significantly higher in ruptured cases, with a mean hemoglobin level of 9.8±2.1 g/dL. Our study also found that 77.2% of cases had moderate-to-severe anemia, reinforcing that delays in diagnosis contribute to significant blood loss and hemodynamic compromise. Studies by Nitesh et al., Dayal and Srivastava, and Yadav et al., similarly reported a higher prevalence of anemia in ruptured cases, indicating that low-resource settings are disproportionately affected by late-stage presentations [18-20].

Biochemical markers, particularly serum β-hCG levels, played a crucial role in determining the severity and progression of EP. Our study found that β-hCG levels >5000 mIU/mL were significantly associated with rupture (OR: 10.01, 95% CI: 2.06-48.69, p=0.004). This aligns with findings from Ferreira et al. and Lu et al., who reported that higher β-hCG levels correlate with increased trophoblastic invasion, leading to more advanced disease and an elevated risk of tubal rupture [21,22]. Additionally, transvaginal ultrasonography (TVS) proved to be an invaluable diagnostic tool, with adnexal mass (63.0%) and free fluid in the pouch of Douglas (65.2%) being the most frequent ultrasonographic findings. These findings are consistent with the previous literature where TVS has demonstrated a sensitivity of 87-90% in diagnosing EP [23,24].

Surgical management was the primary modality of treatment in the present study, with unilateral salpingectomy (43.5%) being the most common procedure performed. The need for blood transfusion was alarmingly high (69.6%), reflecting the severe hemodynamic impact of ruptured cases. Our findings parallel those of Nalini et al., who reported a 94.7% transfusion rate in patients undergoing surgical management for ruptured EP in resource-limited settings [25]. Postoperative complications in our cohort included ICU admission (28.3%), hemoperitoneum >200 mL (30.4%), and wound dehiscence (1.1%), though no maternal mortality was observed. These rates are comparable to a study by Nethra et al., which documented ICU admissions in 30% of cases and a mean hospital stay of 4.8±2.1 days, consistent with our findings [26].

Several predictors of rupture were identified, notably lower socioeconomic status (OR: 4.29, 95% CI: 1.10-16.75, p=0.035), rural residence (OR: 3.69, 95% CI: 0.72-18.98), and hemoglobin <10 g/dL (OR: 10.54, 95% CI: 2.94-37.82, p<0.001). These findings emphasize the intersection between social determinants of health and clinical outcomes. In low-resource settings, socioeconomic disadvantage compounds delays in seeking care, poor antenatal follow-up, and limited emergency access, thereby increasing rupture risk - a finding consistent with studies from Talukder et al. and Uthpala and Gracelyn [27,28]. Interestingly, prior ectopic pregnancy and history of pelvic inflammatory disease were not significantly associated with rupture in our study, contrasting with findings from developed regions where reproductive health profiles differ substantially [29,30].

Limitations

This study has several limitations that should be acknowledged. First, the single-center nature of the study may limit the generalizability of the findings to other populations, especially those with different healthcare infrastructures. Second, while a standardized diagnostic protocol was employed, delays in obtaining ultrasound and β-hCG results in resource-limited settings could have introduced diagnostic variability, potentially affecting the accuracy of early detection. Third, retrospective data collection on certain risk factors, such as prior pelvic inflammatory disease and contraceptive use, may have been subject to recall bias, which could affect the reliability of these findings. Lastly, the study did not assess long-term reproductive outcomes or the psychological impacts on women affected by ectopic pregnancies. Future research should explore the long-term effects of ectopic pregnancies on reproductive health, including the potential for recurrence and fertility preservation. Additionally, studies focusing on the development and implementation of interventions aimed at improving early diagnosis and timely intervention in resource-limited settings would be invaluable in reducing maternal morbidity and improving patient outcomes.

## Conclusions

This study underscores the disproportionately high burden of ruptured ectopic pregnancies in a tribal population, with delayed healthcare access identified as a major contributing factor. Hemodynamic instability, severe anemia, and elevated β-hCG levels were strongly associated with rupture, emphasizing the critical importance of early detection and timely intervention to reduce maternal morbidity. Socioeconomic and geographic disparities significantly influenced maternal outcomes, highlighting the urgent need for targeted public health strategies tailored to underserved populations. Strengthening primary healthcare services, integrating point-of-care diagnostics such as rapid β-hCG testing and ultrasound, and implementing community-based awareness programs are essential steps in improving early detection and minimizing adverse outcomes. Specific public health strategies for tribal populations may include enhancing transportation infrastructure to healthcare facilities, increasing mobile health services, and training local healthcare workers to recognize the signs of ectopic pregnancy. Additionally, fostering collaboration between governmental and non-governmental organizations could facilitate greater resource allocation to these areas. Future research should focus on exploring innovative solutions, such as telemedicine-based ultrasound evaluations, to overcome geographic barriers and bridge healthcare gaps in these underserved regions.

## References

[1] Zhang S, Liu J, Yang L, Li H, Tang J, Hong L (2023). Global burden and trends of ectopic pregnancy: an observational trend study from 1990 to 2019. PLoS One.

[2] Anaswara T, Jacob AT (2021). Clinical profile and risk factors of ectopic pregnancy: a prospective study from a tertiary care center in Kerala. Int J Clin Obstet Gynecol.

[3] Roy AD, Das D, Mondal H (2023). The tribal health system in India: challenges in healthcare delivery in comparison to the global healthcare systems. Cureus.

[4] Flanagan HC, Duncan WC, Lin CJ, Spears N, Horne AW (2023). Recent advances in the understanding of tubal ectopic pregnancy. Fac Rev.

[5] Panelli DM, Phillips CH, Brady PC (2015). Incidence, diagnosis and management of tubal and nontubal ectopic pregnancies: a review. Fertil Res Pract.

[6] Mahajan N, Riana R, Sharma P (2021). Risk factors for ectopic pregnancy: a case-control study in tertiary care hospitals of Jammu and Kashmir. Iberoamerican J Med.

[7] Madankar M, Kakade N, Basa L, Sabri B (2024). Exploring maternal and child health among tribal communities in India: a life course perspective. Glob J Health Sci.

[8] Contractor SQ, Das A, Dasgupta J, Van Belle S (2018). Beyond the template: the needs of tribal women and their experiences with maternity services in Odisha, India. Int J Equity Health.

[9] Cáceres ÁL, Ramesh RM, Newmai P, Kikon R, Deckert A (2023). Perceptions, health seeking behavior and utilization of maternal and newborn health services among an indigenous tribal community in Northeast India - a community-based mixed methods study. Front Public Health.

[10] Mehta D, Kukadiya SB, Guru M, Pandya N (2019). Ectopic tales of Kachchh: a study of ectopic pregnancies [sic]. Int J Clin Obstet Gynaecol.

[11] Soman B, Lathika AR, Unnikrishnan B, Shetty RS (2024). Tracing the disparity between healthcare policy-based infrastructure and health belief-lead practices: a narrative review on indigenous populations of India. J Racial Ethn Health Disparities.

[12] Ahirwar M, Singh P, Dohare R (2023). Clinical study of ectopic pregnancy in tertiary care centre. Trends Clin Med Sci.

[13] Gothwal M, Pathak BL (2018). Ectopic pregnancy-a clinical study. Int J Sci Res.

[14] Andola S, Kumar RR, Desai RM, krutika SA (2021). Study of risk factors and treatment modalities of ectopic pregnancy. J Family Med Prim Care.

[15] Verma ML, Singh U, Solanki V, Sachan R, Sankhwar PL (2022). Spectrum of ectopic pregnancies at a tertiary care center of Northern India: a retrospective cross-sectional study. Gynecol Minim Invasive Ther.

[16] Soren M, Patnaik R, Sarangi BK (2017). A clinical study on ectopic pregnancy. Int J Res Med Sci.

[17] Barik S, Malakar A, Laha S (2020). Trends in ectopic pregnancy: a prospective observational study from a tertiary care center in Eastern India. J South Asian Feder Obstet Gynecol.

[18] Nitesh M, Bairwa R, Sharma S (2020). Study of ectopic pregnancy in a tertiary care centre. Int J Reprod Contracept Obstet Gynecol.

[19] Dayal N, Srivastava A (2019). A retrospective study on ectopic pregnancy in a tertiary care hospital. IOSR J Dent Med Sci.

[20] Yadav DP, Bhati I, Bhati BS (2016). Ectopic pregnancy: a comprehensive analysis of risk factors and management. Int J Reprod Contracept Obstet Gynecol.

[21] Ferreira DF, Elito Júnior J, Araujo Júnior E, Stavale JN, Camano L, Moron AF (2014). Trophoblastic infiltration in tubal pregnancy evaluated by immunohistochemistry and correlation with variation of beta-human chorionic gonadotropin. Patholog Res Int.

[22] Lu J, Yue X, Xu C, Lu X (2017). Primary gestational trophoblastic disease of the fallopian tube: a case series analysis and literature review. J Reprod Med.

[23] Honemeyer U (2019). Three-dimensional ultrasound imaging in the diagnosis of ectopic pregnancy. Donald Sch J Ultrasound Obstet Gynecol.

[24] Nadim B, Infante F, Lu C, Sathasivam N, Condous G (2018). Morphological ultrasound types known as 'blob' and 'bagel' signs should be reclassified from suggesting probable to indicating definite tubal ectopic pregnancy. Ultrasound Obstet Gynecol.

[25] Nalini N, Singh KA, Neetu S, Kumari A (2023). Clinical profile, risk factors and outcomes of ectopic pregnancy in a tertiary care hospital: a prospective Indian study. Cureus.

[26] Nethra HS, Praneetha K, Sreelatha S, Bhairi SS (2018). A study on risk factors and clinical presentation of ectopic pregnancy. New Indian J OBGYN.

[27] Talukder A, Burman SK, Bera G, Mukherjee J, Maji M, Santra D (2023). A cross-sectional observational study of the etio-pathology, socioeconomic distribution and clinical pictures of ectopic pregnancy in a Tertiary Medical College and Hospital in Bankura, West Bengal, India. J Med Sci Health.

[28] Uthpala V, Gracelyn JL (2022). Study of risk factors associated with ectopic pregnancy: an observational study. Int J Reprod Contracept Obstet Gynecol.

[29] Xu C, Mao Z, Tan M (2023). Prevalence and related factors of rupture among cases with ectopic pregnancy; a systematic review and meta-analysis. Arch Acad Emerg Med.

[30] Wang X, Huang L, Yu Y, Xu S, Lai Y, Zeng W (2020). Risk factors and clinical characteristics of recurrent ectopic pregnancy: a case-control study. J Obstet Gynaecol Res.

